# “Stem cell therapy to promote limb function recovery in peripheral nerve damage in a rat model” – Experimental research

**DOI:** 10.1016/j.amsu.2019.03.009

**Published:** 2019-03-28

**Authors:** Jason R. Bingham, Kevin R. Kniery, Nikolas L. Jorstad, Iren Horkayne-Szakaly, Zachary S. Hoffer, Shashikumar K. Salgar

**Affiliations:** a Department of Surgery, Madigan Army Medical Center, Tacoma, WA, 98431, USA; b Department of Pathology, University of Washington, Seattle, WA, 98195, USA; c Department of Neuropathology & Ophthalmic Pathology, Joint Pathology Center, Defense Health Agency, Silver Spring, MD, 20910, USA; d Department of Pathology, Madigan Army Medical Center, Tacoma, WA, 98431, USA; e Department of Clinical Investigation, Madigan Army Medical Center, Tacoma, WA, 98431, USA

**Keywords:** Mesenchymal stem cells, Sciatic nerve repair, Microsurgery, Rat, Cell therapy, Motor function

## Abstract

**Background:**

Optimizing nerve regeneration and mitigating muscle atrophy are the keys to successful outcomes in peripheral nerve damage. We investigated whether mesenchymal stem cell (MSC) therapy can improve limb function recovery in peripheral nerve damage.

**Materials and methods:**

We used sciatic nerve transection/repair (SNR) and individual nerve transection/repair (INR; branches of sciatic nerve - tibial, peroneal, sural) models to study the effect of MSCs on proximal and distal peripheral nerve damages, respectively, in male Lewis rats. Syngeneic MSCs (5 × 10^6^; passage≤6) or saline were administered locally and intravenously. Sensory/motor functions (SF/MF) of the limb were assessed.

**Results:**

Rat MSCs (>90%) were CD29^+^, CD90^+^, CD34^−^, CD31^−^ and multipotent. Total SF at two weeks post-SNR & INR with or without MSC therapy was ∼1.2 on a 0–3 grading scale (0 = No function; 3 = Normal); by 12 weeks it was 2.6–2.8 in all groups (n ≥ 9/group). MSCs accelerated SF onset. At eight weeks post-INR, sciatic function index (SFI), a measure of MF (0 = Normal; −100 = Nonfunctional) was −34 and −77 in MSC and vehicle groups, respectively (n ≥ 9); post-SNR it was −72 and −92 in MSC and vehicle groups, respectively. Long-term MF (24 weeks) was apparent in MSC treated INR (SFI -63) but not in SNR (SFI -100). Gastrocnemius muscle atrophy was significantly reduced (P < 0.05) in INR. Nerve histomorphometry revealed reduced axonal area (P < 0.01) but no difference in myelination (P > 0.05) in MSC treated INR compared to the naive contralateral nerve.

**Conclusion:**

MSC therapy in peripheral nerve damage appears to improve nerve regeneration, mitigate flexion-contractures, and promote limb functional recovery.

## Highlights

• Mesenchymal stem cell (MSC) therapy improved limb functional recovery.
• MSCs improved nerve regeneration and mitigated foot flexion-contractures.
• Limb muscle atrophy was significantly reduced in individual nerve repair (INR).
• Functional recovery in distal nerve repair (INR) was superior to proximal (SNR).
• MSC therapy is attractive, feasible & promising in peripheral nerve injury repair.

## 1 Introduction

Functional recovery is of utmost importance for limb salvage in the management of peripheral nerve injuries. These injuries have been reported to affect 2.8% of trauma patients [1]. Injuries can range from compression of the nerve to complete nerve transection with no continuity of any neural structure. In the latter case, surgical re-anastomosis is the only reliable method of treatment. Trauma can affect the sciatic, femoral, facial and other peripheral nerves causing respective regional paralysis. Sciatic nerve injury, common peripheral neuropathy is characterized by muscle weakness, reflex changes, and numbness. The majority of patients complain of persistent and severe pain, motor dysfunction and prolonged disability [2]. Efforts to restore muscle function are compromised by the slow growth rate of the nerve axon which delays muscle re-innervation [3].

Despite advances in epineural or perineural sutures for tension-free nerve repair, the outcome is still sub-optimal; this may be due to many factors, both intrinsic and extrinsic to the nervous system [4]. There are several alternative approaches under investigation, including stem cell transplantation [3,[5], [6], [7], [8], [9]]. The role of implanted stem cells on peripheral nerve regeneration is not completely understood. However, it has been suggested that a combination of several features such as trophic factor production, extracellular matrix synthesis, axon guidance and sorting, remyelination, micro environmental stabilization, and immune modulation support peripheral nerve regeneration and function [7,8,[10], [11], [12], [13], [14], [15], [16], [17], [18], [19]]. Poor sensory and motor functional recovery is due, in part, to the suboptimal regeneration of transected peripheral nerve components and re-innervation of target muscle groups [[20], [21], [22]]. One way to enhance nerve regeneration is by using adult mesenchymal stem cells (MSCs) that have the potential to self-renew and differentiate into several lineages including neuronal cell types such as Schwann cells [23,24]. Therapeutic benefits of MSCs have been shown in animal models of Parkinson's disease, multiple sclerosis, stroke, traumatic brain injury, spinal cord injury, and peripheral nerve damage [[25], [26], [27], [28]]. MSCs have the potential to induce myogenesis and angiogenesis by releasing different angiogenic, mitogenic, and anti-apoptotic factors including VEGF, IGF-1, HGF, and Bcl-2(29). Also, MSCs produce other paracrine factors such as heat shock protein 20 (HSP20), hemeoxygenase-1 (HO-1), stem cell factor (SCF) and stromal cell derived factor (SDF) which are involved in remodeling, regeneration, and neovascularization, leading to improvement in organ function [29]. MSCs have been shown to improve blood flow in a rat hind-limb ischemic model, due to their paracrine factors such as VEGF, TGF-β1 and NO [30]; and have the unique ability of migrating to areas of hypoxia and tissue injury, and augmenting tissue repair [[31], [32], [33]].

MSCs can be administered topically or systemically. Topical administration presents an advantage that MSCs arrive directly to the site of lesion (target organ) referred to nonsystemic homing [34]. With intravenous administration, cells are easily trapped in lung, liver or spleen because of their larger size and expression of adhesion molecules like integrin CD49f or CD49d which results in reduced number of cells (∼2%) delivered to target site [35,36]. However, circulating MSCs preferentially migrate, extravasate at the lesion vicinity, and accumulate at sites of tissue damage and inflammation; this is in response to chemoattractants particularly stromal cell derived factor 1 (SDF-1) which interacts with the CXCR4 receptor expressed on MSC [37,38] and is referred to systemic homing [34]. Efficient homing and migration of MSCs towards lesion sites play an important role in MSC therapy.

The objectives of this study were: 1) To determine whether MSC therapy can improve limb functional recovery in peripheral nerve damage; and 2) To determine whether there is any difference in limb functional recovery, nerve regeneration and target muscle atrophy between proximal and distal peripheral nerve damage with or without MSC therapy.

## 2 Material and methods

### 2.1 Animals

We used inbred male Lewis (RT1^l^) rats, ten- to 12-week-old weighing ∼300 g, purchased from Harlan Sprague Dawley (Indianapolis, IN). The rationale for using all male rats was to avoid hormonal influence which varies with the reproductive phase in females. Animals were maintained according to the 'Guide for the Care and Use of Laboratory Animals' published by the National Research Council/Institute of Laboratory Animal Research (ILAR). All animal housing, husbandry and experiments were conducted following approval by our Institutional Animal Care and Use Committee (IACUC), as per protocol and institutional guidelines. The research being reported is in accordance with the ARRIVE (Animal Research: Reporting In Vivo Experiments) [39].

### 2.2 Experimental design

Our study included four experimental groups (n = 9–12/group). Group A. Sciatic Nerve Repair (SNR) model, received saline (vehicle); Group B. SNR model, received MSC; Group C. Individual Nerve Repair (INR) model, received saline (vehicle); and Group D. INR model, received MSC. The SNR involved transection and repair of the main sciatic nerve branch (proximal nerve damage), while INR included transection of the distal branches of the sciatic nerve (tibial, sural, and peroneal) and repair (distal nerve damage). Nerve transection and repair was done on the right hind limb, and the contralateral limb served as a non-transected (naïve) nerve control. Starting ≥1 week post-SNR or INR, animals received manual physiotherapy for the right hind limb (≤5 min, 1–2 times per week) as described previously [40]. Primary outcome measures were limb sensory and motor functions, and secondary outcome measures were gastrocnemius mass, flexion contractures, and nerve histology.

### 2.3 MSC preparation and administration

**Fig. 1:**
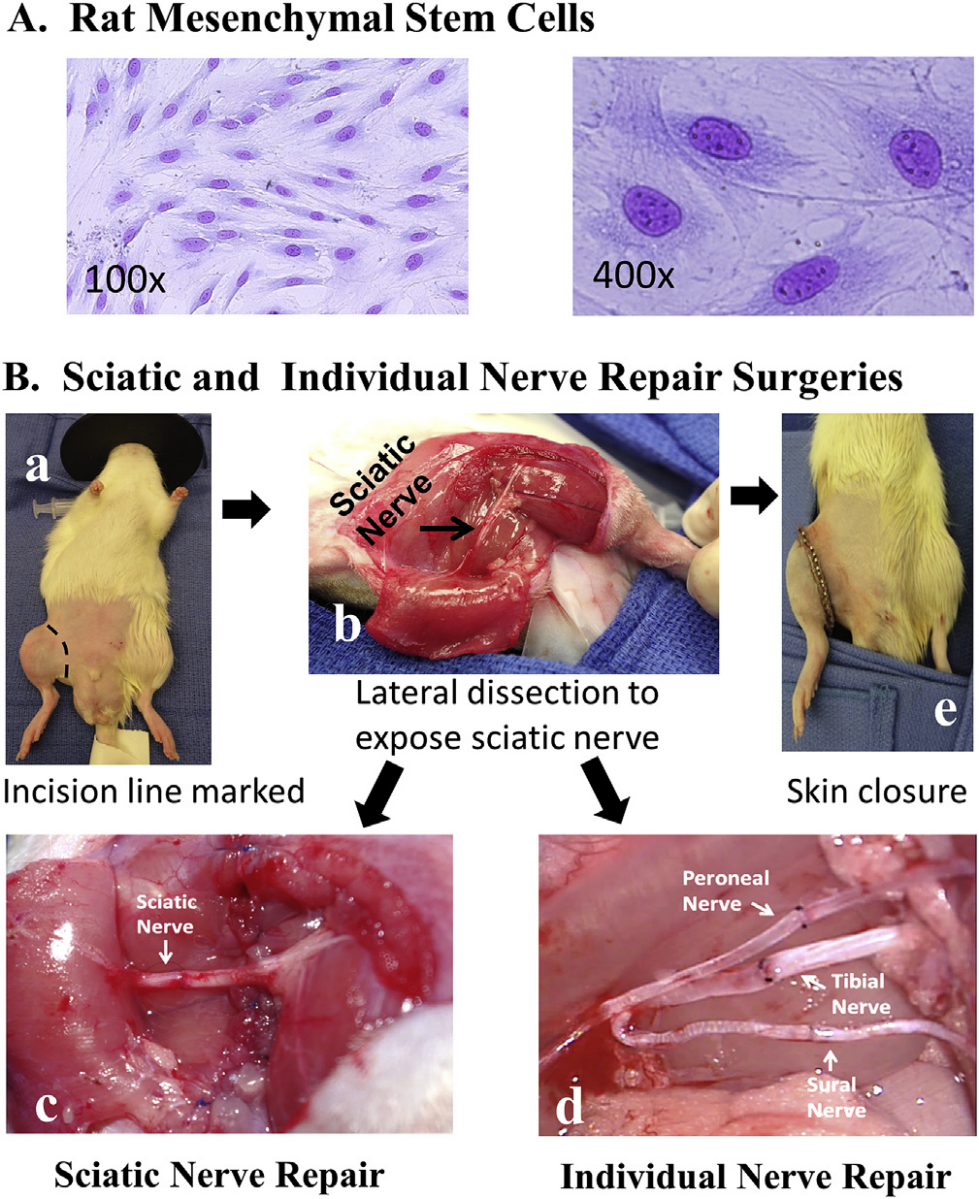
*A, Mesenchymal Stem Cells (MSC) stained with Wright's stain (100X and 400X). B, Sciatic and individual nerve repair surgeries*: a, animal prepared for right hind limb dissection for sciatic nerve transection and repair surgeries; b, lateral dissection to expose sciatic nerve; c, sciatic nerve transected and repaired with interrupted sutures; d, individual branches of sciatic nerve (peroneal, tibial, sural) were transected and repaired similar to sciatic nerve repair; and e, the muscles were approximated and skin incision was closed.

We isolated, expanded, and administered MSCs (Fig. 1 A) as previously described [40].

Briefly, Lewis (RT1.A^l^) rats were euthanized by injecting pentobarbitol (40–80 mg/rat) based solution intraperitoneally. Long bones (tibia, femur) were harvested aseptically and transferred to the laboratory quickly (<30 s) in 70% alcohol. Following thorough rinsing of bones in sterile phosphate buffered saline, bone marrow cells (BMCs) were isolated from the bones, and suspended at 5–10 × 10^7^ cells/ml in MSC complete or growth medium. The complete medium was prepared using Dulbecco's Modified Eagle's medium [DMEM] - Low glucose, Glutamax, Pyruvate, 10% fetal bovine serum, penicillin [100 units/ml], and streptomycin [100 mg/ml]; all reagents were obtained from Gibco/Life Technologies, NY. BMCs isolated were plated (0.5 × 10^6^ cells/cm^2^) in 75 or 175 cm^2^ flasks and cultured at 37 °C in 5% CO2 in complete medium; about 72 h following culture, non-adherent cells (floating) in the supernatant were removed completely and the medium was replaced with fresh complete medium. Adherent cells were cultured for an additional 2–4 weeks; when the cultures were ∼70% confluent, they were sub-cultured (1:3). Early passage (≤3) MSCs were harvested and frozen in RPMI 1640 medium containing 10% dimethyl sulfoxide (DMSO), 30% fetal bovine serum, penicillin (100U/ml) and streptomycin (100 μg/ml). The cells were stored at −150 °C for future use. About one to two weeks prior to MSC injection, frozen cells were removed, quickly thawed in a water bath (37 °C) and expanded in cultures using complete medium.

Briefly, following surgical nerve repair, MSCs (5 × 10^6^ cells per animal in ∼0.5 ml saline) or vehicle (saline) were infused locally (at nerve repair sites) before muscle approximation and skin closure. Immediately after surgery, MSCs (5 × 10^6^ cells per animal in 1–1.5 ml of saline) or vehicle was injected intravenously (IV) via the dorsal penile vein. Intravenous MSC (5 × 10^6^ cells) or vehicle injections were repeated at weekly intervals for three additional weeks.

To ensure adequate number of MSCs home lesion site we administered both locally and systemically. The MSC dose and frequency used in this study was consistent with our own previous report [40] where we performed hind limb transplants in rats that involved sciatic nerve transection and repair followed by the administration of MSCs. Also, our protocol was similar to Yang et al. [41] where they studied dual regeneration of muscle and nerve by intravenous injection of human-amniotic fluid-derived mesenchymal stem cells (5 × 10^6^ for 3 days daily following surgery) in a sciatic nerve injury model. In a mouse model of sciatic crush injury, Marconi and co-workers [17] injected 2 × 10^6^ human-adipose derived mesenchymal stem cells (hMSC) 7 days after surgery and observed improved limb function recovery. We believe the dose and frequency of MSCs administered in the present study were appropriate and based on previous reports.

### 2.4 Surgical procedures

The general surgical techniques used for SNR and INR are described previously [40,42,43] and the specific procedures used in this study are as follows.

#### 2.4.1 Sciatic nerve repair model

The sciatic nerve repair surgical procedure is shown in Fig. 1B. We anesthetized rats by injecting Ketamine (40–80 mg/kg) and Xylazine (5–10 mg/kg) intraperitoneally; and anesthesia was maintained with inhalant 1–2% isoflurane. The animal received a preoperative antibiotic, cefazolin (25 mg/kg body weight) subcutaneously (SQ), and the eyes were lubricated with ophthalmic ointment (Vidisic) to prevent corneal drying. The surgical site was depilated using clippers, and sterilized with 10% chlorhexidine and 70% alcohol. Body temperature (∼38 °C) was maintained by placing the animal on a thermos regulated warming pad. A skin incision around the right thigh (at the level of inguinal ligament) was made and the skin was mobilized to expose biceps femoris. The biceps femoris was then divided near the distal attachments to the stifle and tibia and reflected to expose the sciatic nerve which was then dissected out proximally to the point of emergence from below the gluteus muscle. The sciatic nerve was transected proximal to the trifurcation of the tibial, peroneal, and sural nerves after tag sutures of 10-0 nylon was placed on proximal and distal ends of the transected nerve. Heparin 50U in 300 μl was administered via the tail vein for anti-coagulation. After one hour of wait time (to mimic surgical or injury situation), Neurorrhaphy (sciatic nerve ends were approximated) was performed with 10-O nylon sutures, followed by biceps femoris repair and skin closure.

#### 2.4.2 Individual nerve repair model

The procedure was similar to the SNR described above. However, in INR, the branches of the sciatic nerve (tibial, peroneal, and sural) were dissected out, transected, and surgically repaired (Fig. 1B). The proximal and distal ends of the sciatic nerve or individual nerves were prepared by removing excess mesoneurium, exposing the cut edges of epineurium. After orientation by aligning the fascicles, the epineurium was approximated in a tension-free manner using 2–4 interrupted sutures of 10-0 nylon in both SNR and INR models. MSCs or vehicle was administered topically at the sites of nerve repair. The dissected biceps muscle was sutured using 6-0 prolene in a running locking fashion. The skin incision was closed using 4-0 nylon interrupted sutures and stainless steel clips.

#### 2.4.3 Postoperative animal management

Post-operative care and physiotherapy were provided as described previously [40,43]. To prevent dehydration Lactated Ringers solution was administered (5 cc, SQ); as analgesic, buprenorphine (0.02–0.05 mg/kg, SQ) was administered every 12 h as needed; and cefazolin (20 mg/kg, SQ) was administered every 12 h for 3 days. Body weights were monitored daily/weekly and animals were closely observed for signs of pain or distress. Animals received physiotherapy 1–2 times per week (5 min/session), beginning 1–2 weeks post-surgery. Briefly, nerve repaired limb was gently and repeatedly manipulated through the normal range of motion under manual restraint as described previously [43]. Each physiotherapy session lasted up to 5 min, as long as the animal tolerated it well. For additional physiotherapy, animals were allowed to stay in wire floor mesh group housing cage (equipped with solid floor space access with in the cage) for about 8 h a day.

### 2.5 Evaluation of limb function

#### 2.5.1 Sensory function assessment

**Fig. 2:**
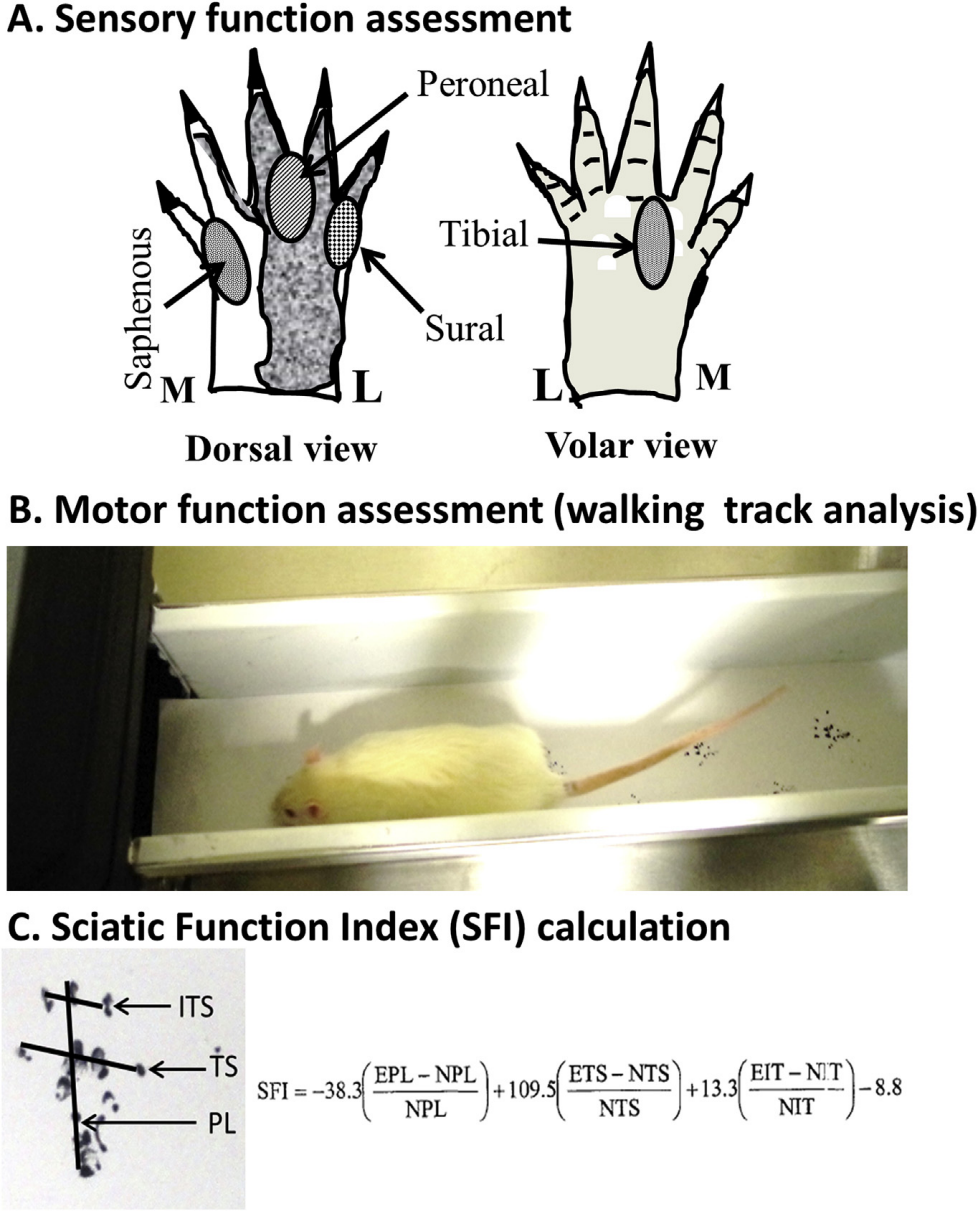
*Limb functional analysis.* A. Sensory function assessment (Cutaneous Pain Reaction Test): Sensory function was assessed by pinch technique in the territories of the tibial (T), peroneal (P), sural (Sur), and saphenous (S) nerves as described, previously [39, 40]. Withdrawal/vocal response was scored in comparison to the normal limb (0 = no response, 1 = slight, 2 = moderate, 3 = normal). L, Lateral; M, Medial. B, Motor function assessment (Walking Track Analysis): *Progression of walking track* of naive and nerve transected/repaired limb foot-prints were obtained on a paper in a rat walking apparatus as described previously [39, 45–48] C, Sciatic Function Index (SFI) was calculated from the following foot-print measurements (mm): EPL, Experimental plantar length; NPL, Normal plantar length; ETS, Experimental toe spread; NTS, Normal toe spread; EIT, Experimental intermediary toe spread; NIT, Normal intermediary Toe Spread; PL, Plantar length (distance between heal to middle 3rd toe); TS, Toe spread (distance between 1st and 5th toe); IT, 2nd and 4th toe distance; ITS, Intermediary toe spread.

Cutaneous pain reaction test also called *the flexor* “*withdrawal*” spinal reflex test was used as previously described [40,43]. Normal innervation results in an immediate withdrawal response, with or without vocalization. We tested animals for sensory function beginning one-two weeks post nerve repair and continued at weekly intervals. Briefly, animals were handheld with the hind-limbs in suspension, and allowed to relax. Using atraumatic forceps the stimulus was applied briefly by pinching in selected areas innervated by the tibial, peroneal, sural and saphenous nerves (Fig. 2A) as previously described [43,44]. The stimulus was first applied to the normal (left) hind limb, and the response was graded and recorded. The stimulus was then applied in the same nerve boundary area of the nerve repaired hind-limb (right), and the response was graded in comparison to the contralateral normal limb. The withdrawal reflex was graded as described previously [45]: 0, No response; 1, Mild response; 2, Moderate response; 3, Strong response (normal). Note animals were not sedated or anesthetized for this analysis.

#### 2.5.2 Motor Function Assessment

Walking track analysis was used as described previously [40,[46], [47], [48], [49]]. Briefly, a confined walkway (10 cm wide x 10 cm high x 70 cm long), lined with white paper and led into a dark shelter was used (Fig. 2B). Black ink (water soluble) was applied to the plantar surfaces of the hind feet, and the animal was allowed to walk down the corridor into the shelter. Animals were conditioned by practice trials 3–5 days prior to nerve transection and repair surgery. Walking track analysis began two weeks post-nerve repair and continued at two-week intervals. We calculated the sciatic function index (SFI), a conventional measure to assess hind limb motor function, using foot print characteristics as described previously (Fig. 2C) [46].

### 2.6 Gastrocnemius muscle mass

After animals were euthanized, the gastrocnemius muscles of the nerve repaired (right) and contralateral native (left) hind limbs were dissected, harvested, and weighed. The mean muscle weights were compared between normal (left) and nerve repaired (right) limbs in each group, between groups (vehicle & MSC), and between the models (SNR and INR).

### 2.7 Histology and histomorphometry

We harvested nerve segments of about 5–10 mm length five millimeters distal to the nerve transection/repair site, preserved in 3% glutaraldehyde, post-fixed in osmium tetroxide, embedded in plastic, cut into one-micron thick cross sections, stained with toluidine blue and evaluated by light microscopy for axonal variation as described previously [50]. The toluidine blue-stained sections were evaluated by light microscopy. Images (400x) were used for all morphometric analyses and were processed in *ImageJ* (NIH, Bethesda, MD) to determine the axonal size (area) and *g* ratio (axonal myelination). The *g* ratio is the ratio between the diameter of the axon and the outer diameter of the myelinated fiber.

### 2.8 Statistical analysis

The SPSS software version PASW Statistics18 (SPSS Inc., Chicago, IL) was used for statistical analyses. The data between the two groups were compared by Student *t*-test or ANOVA with Bonferroni correction. All P-values were two-tailed, and values ≤ 0.05 were considered statistically significant.

## 3 Results

### 3.1 Animal body weight

The body weight between vehicle and MSC treated rats in SNR and INR groups did not differ significantly (P > 0.05; Table S1).

### 3.2 Mesenchymal stem cell characteristics

Greater than 90% *ex vivo* cultured MSCs (passage ≤6) expressed MSC markers (CD29, CD90) and less than 10% expressed Hematopoietic Stem Cell markers (CD31, CD34, CD45). MSCs were pluripotent as determined by osteogenesis, adipogenesis, and chondrogenesis differentiation assays in a parallel study [40].

### 3.3 Limb functional recovery

The SNR and INR surgeries performed in this study are shown in Fig. 1B.

#### 3.3.1 Sensory function

**Fig. 3:**
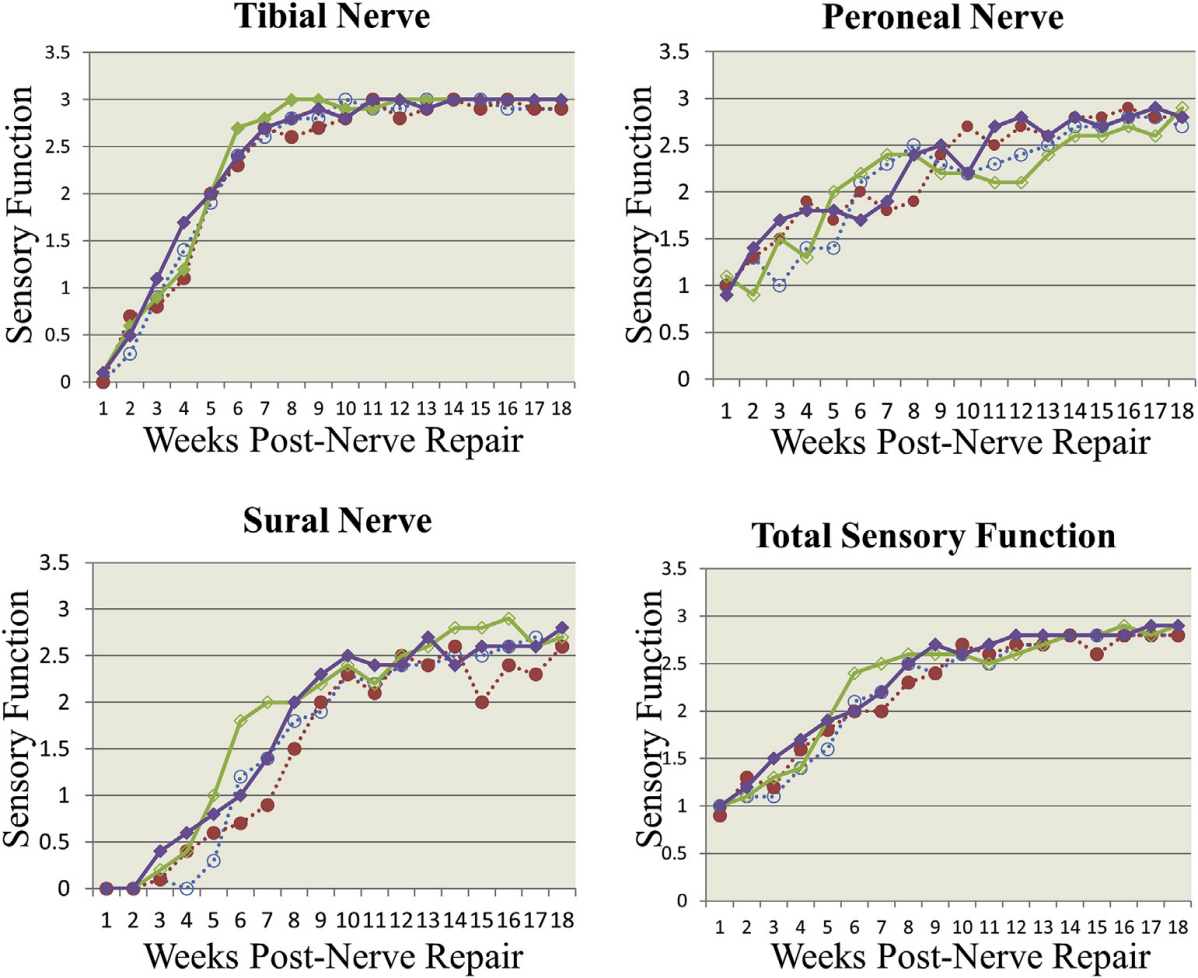
*Sensory Function Assessment:* Sensory function was assessed by pinch technique in the territories of the tibial, peroneal, sural and saphenous nerves as described, previously [39, 40] Withdrawal/vocal response was scored in comparison to the naive contralateral limb (0 = no response, 1 = slight, 2 = moderate, 3 = normal). The sensory score was assessed in individual nerve repair (INR) and sciatic nerve repair (SNR) models in the vehicle (control) and mesenchymal stem cell (MSC) treatment groups up to 18 weeks post-nerve repair. Peroneal nerve function was recovered first followed by tibial and sural nerves. Total (overall) sensory function which is the average of all four nerve boundaries (peroneal, tibial, sural and saphenous) was not significantly different (P > 0.05) between vehicle and MSC treated groups. However, sensory function recovery was slightly better in the MSC group than in the vehicle group. Solid square = INR MSC treatment (n = 10); Empty square = INR Vehicle control (n = 12); Solid circle = SNR MSC treatment (n = 9); and Empty circle = SNR Vehicle control (n = 12).

The mean sensory function scores are presented in Table S2 and Fig. 3. The sensory function recovery was earliest in the peroneal nerve territory (∼1 week), followed by the tibial (∼2 weeks), and sural (∼6 weeks) in both SNR and INR models. Total sensory function at two weeks post SNR or INR with or without MSC therapy ranged from 1.1 to 1.2 on a scale of Grade 0–3 (0 = No function; 3 = Normal). However, at four weeks post-nerve repair, total sensory nerve function in SNR model was grade 1.39 ± 0.37, and 1.53 ± 0.45 in vehicle and MSC treated animals, respectively; in the INR model, it was 1.36 ± 0.27 and 1.62 ± 0.46, respectively. By eight weeks it was significantly (P < 0.05) higher (Grade 2.2 to 2.6) compared to four weeks (Table S2). The overall (total) sensory functional recovery improved over time ∼ Grade 2.7 by 18 weeks (n ≥ 10/group).

#### 3.3.2 Motor function

**Fig. 4:**
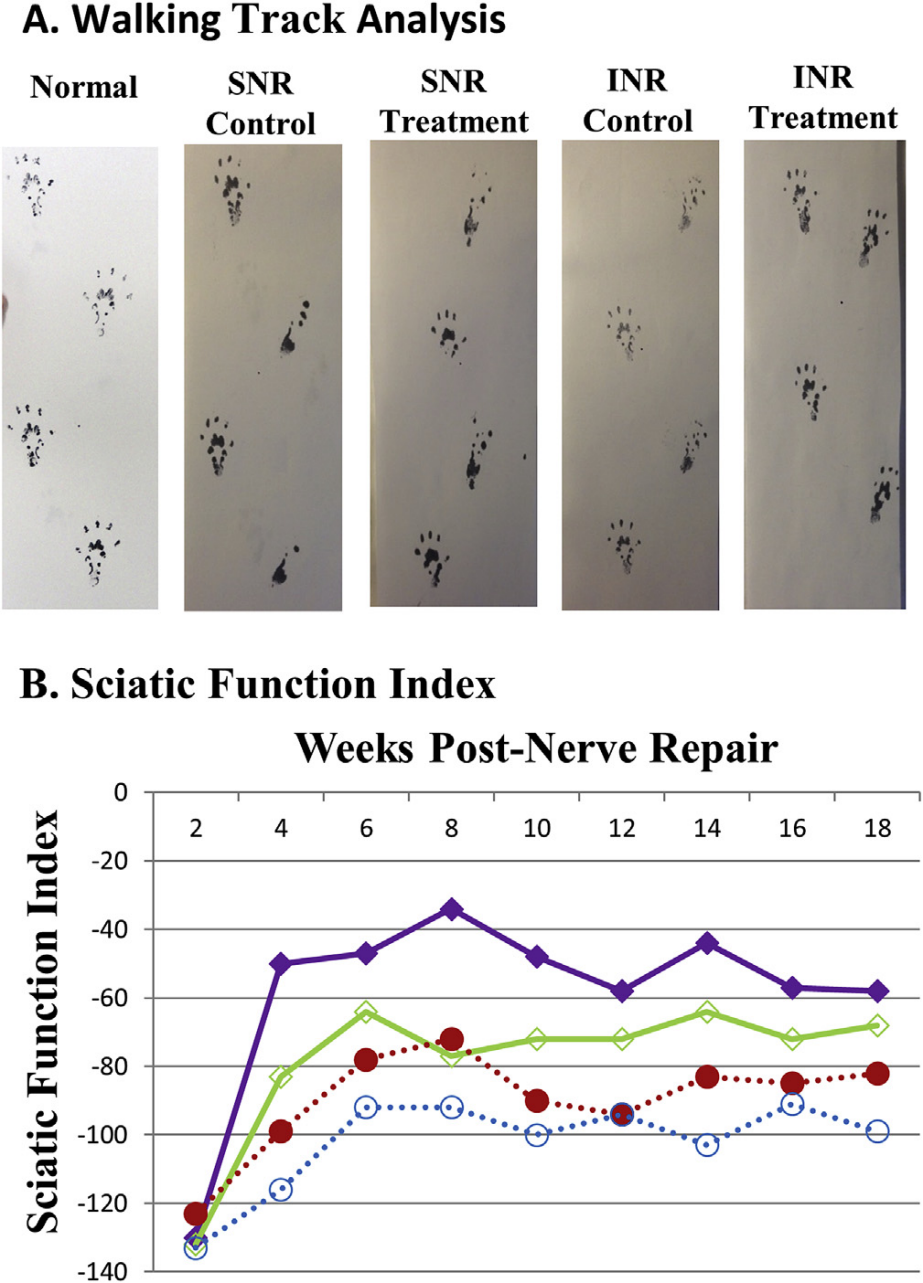
*Motor Function Assessment.* A. Walking track analysis: progression of walking track (foot prints) of normal (naïve), and nerve transected/repaired animals in vehicle (Control) and mesenchymal stem cell (MSC) treated (Treatment) groups in individual nerve repair (INR) and sciatic nerve repair (SNR) models up to 18 weeks post nerve repair are shown. In INR with MSC treatment footprints improved significantly compared to the vehicle control group; INR footprints were better than the SNR footprints. B, Sciatic Function Index: a measure of the motor function calculated from footprints (see materials and methods) was significantly improved (P < 0.05) in INR with MSC treatment compared to vehicle-treated control. Also, SFI was markedly improved in SNR with MSC treatment compared to vehicle-treated control. Interestingly, SFI (footprints) in INR was significantly better than SNR. Solid square = INR MSC treatment (n = 10); Empty square = INR Vehicle control (n = 12); Solid circle = SNR MSC treatment N = 9); and Empty circle = SNR Vehicle control (n = 12).

The walking track prints and SFI are shown in Fig. 4 A&B. At eight weeks post-INR, the SFI (0 = Normal; −100 = Nonfunctional) was −34 and −77 in MSC and vehicle groups, respectively (n ≥ 10); in SNR it was −72 and −92 in MSC and vehicle groups, respectively(n ≥ 9). Long-term motor function (24 weeks) was apparent in MSC treated INR model (SFI -63) but not in SNR model (SFI -100). Motor function was significantly (P < 0.05) improved in INR model compared to SNR model; MSC treatment further enhanced motor function.

### 3.4 Gastrocnemius muscle mass

In SNR model treated with vehicle or MSC, gastrocnemius muscle mass of the nerve repaired limb was significantly (P < 0.05) reduced (33–35%) compared to the contralateral naïve limb. In INR model treated with vehicle or MSC, the nerve repaired limb gastrocnemius muscle mass was reduced only by 14–16% compared to the contralateral naïve limb (Table S1).

### 3.5 Foot-flexion contractures

In the SNR model, 75% (9 out of 12) and 77% (7 out of 9) animals developed flexion contractures in vehicle and MSC treated groups, respectively. In the INR group, 33% (4 out of 12) and 20% (2 out of 10) animals developed flexion contractures in vehicle and MSC treated groups, respectively.

### 3.6 Histology and histomorphometry

**Fig. 5:**
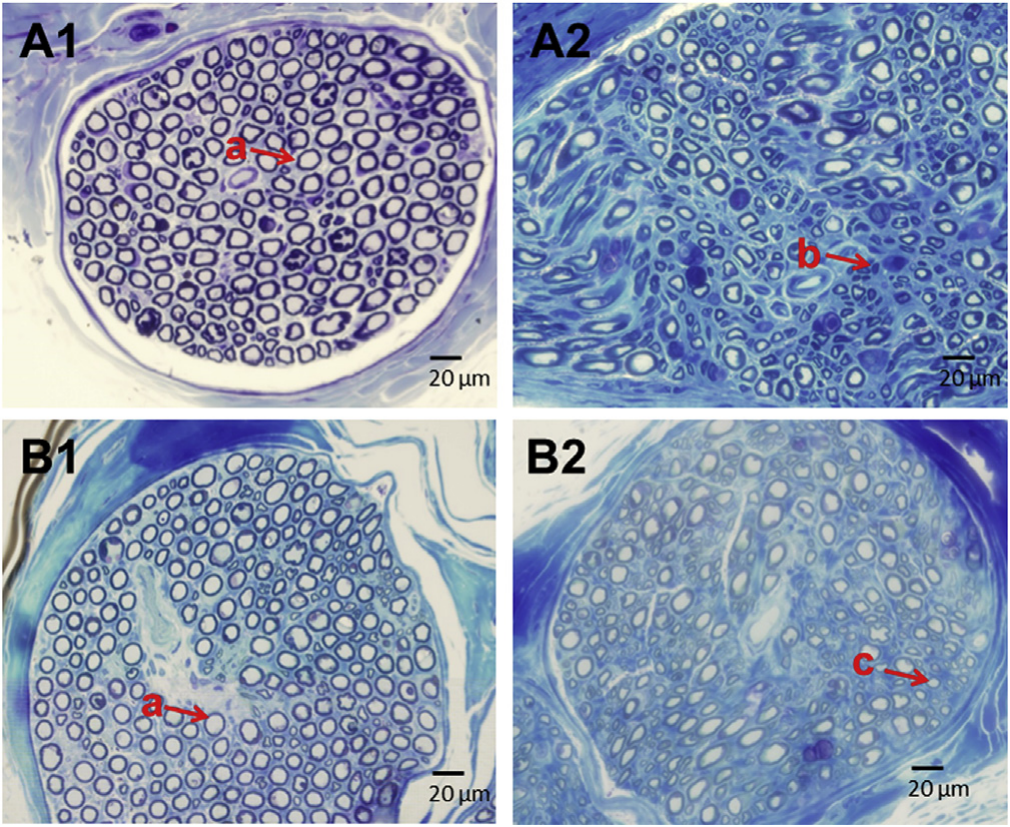
*Histology of tibial nerve in individual nerve repair (INR) model*. Tibial nerve distal to the nerve transection repair site was harvested, cut into semi-thin (1 μm) sections and stained with Toluidine Blue. Cross section images (400x) were used for comparison by light microscopy. *A1, Left tibial naïve nerve untransected and vehicle-treated -* numerous axons tightly packed and surrounded by distinct myelin sheaths; *A2, Right tibial transected and repaired nerve vehicle-treated -* fewer axons, less distinct, reduced myelination, reduced nerve fiber density, increased axonal degeneration, and some regenerating axons (small diameter). B1, *Left tibial naïve nerve untransected and MSC treated* – numerous axons tightly packed and surrounded by distinct myelin sheath as in A1; B2, *Right tibial transected and repaired nerve MSC treated* – increased large size axons, nerve fiber density, myelination and regenerating axons, and reduced axonal degeneration compared to vehicle-treated control A2. a, normal axons (axons visible as blue annulae with myelination evident); b, degenerating axons (axons less distinct, myelination decreased, and myelin debris evident); c, regenerating axons (axons visible with distinct myelination and more nerve fiber density); MSC, mesenchymal stem cells. (For interpretation of the references to colour in this figure legend, the reader is referred to the Web version of this article.)

Histology of tibial nerve sections from INR model is shown in Fig. 5. Left tibial nerve untransected (naïve) treated with vehicle (saline) showed normal features (Fig. 5A1): numerous tightly packed large myelinated fibers and endoneurial blood vessels. Right, tibial nerve transected, repaired and treated with vehicle (saline) demonstrated characteristic features of nerve injury (Fig. 5A2): fewer axons, less distinct and fewer large myelinated fibers, reduced nerve fiber density, increased axonal degeneration, some axonal atrophy and occasional regenerating axon clusters. Left tibial nerve untransected (naive) but treated with MSC (Fig. 5B1) showed normal features similar to Fig. 5A1. Right, tibial nerve transected, repaired, and treated with MSC (Fig. 5B2) showed an increased number of distinct axons, larger myelinated fibers, increased nerve fiber density, increased number of regenerating axons, and reduced axonal degeneration compared to Vehicle treated control group (Fig. 5A2).

**Fig. 6:**
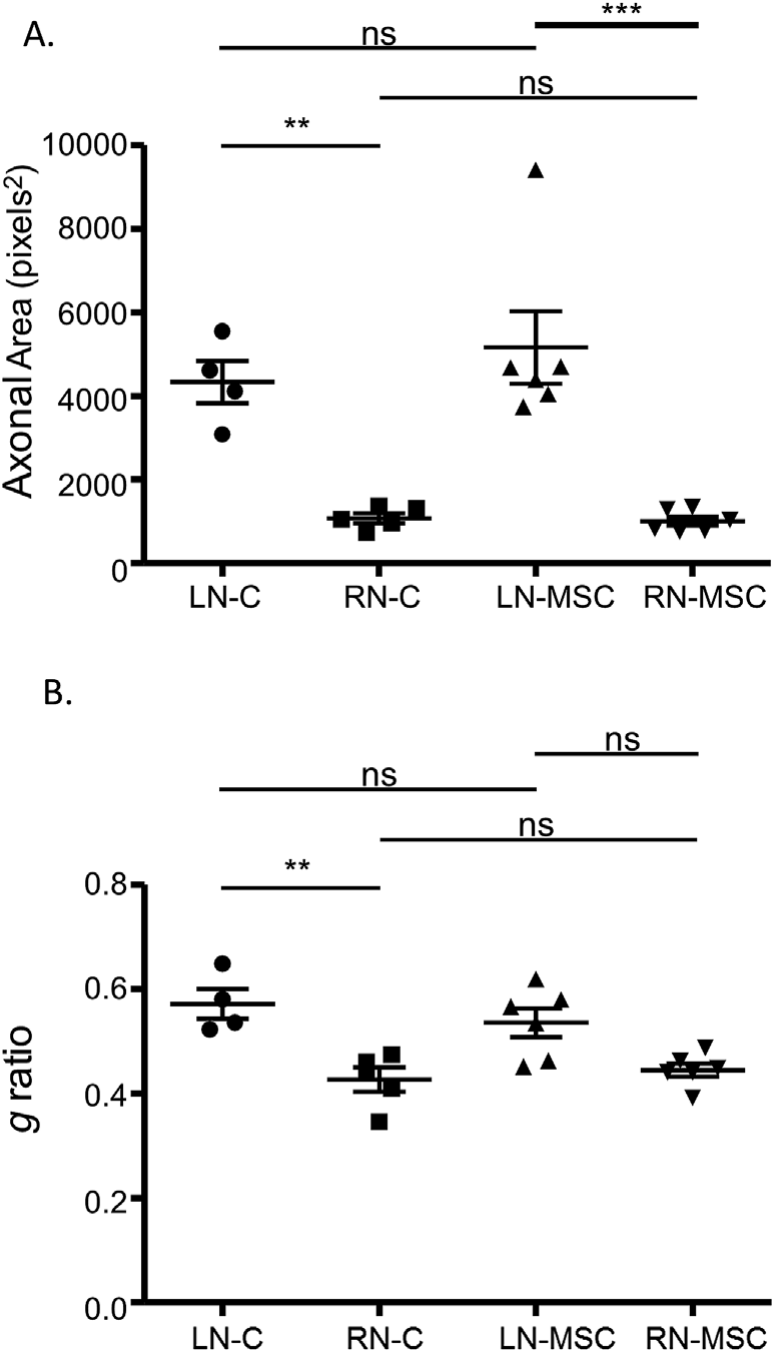
*Histomorphometry of the tibial nerve in individual nerve repair model*. Toluidine Blue stained nerve cross-section images (400x) were processed using ImageJ software (NIH, Bethesda, MD) to determine axonal size and myelination. A. Axon diameter/size (area): significant decrease in axonal area in the right repaired nerve (RN) compared to contralateral naïve left nerve (LN) in both control (C) (P < 0.01) and MSC (P < 0.001) treated groups were observed. B, Axon myelination (*g* ratio): in vehicle-treated control group, myelination (*g* ratio) was significantly (P < 0.01) reduced in the right repaired nerve (RN-C) compared to the contralateral naïve left nerve (LN-C). In MSC treated group there was no significant difference (P > 0.05) in myelination (*g* ratio) between the naïve left nerve (LN-MSC) and repaired right nerve (RN-MSC). ns, non-significant, MSC, Mesenchymal Stem Cells; *P < 0.05, **P < 0.01, ***P < 0.001.

Comparative histomorphometry of the tibial nerve in INR model is shown in Fig. 6. The right tibial repaired nerve in Vehicle treated control animals (RN-C) showed a significant reduction in axonal area (P ≤ 0.01; Fig. 6A) and reduction in *g* ratio (axonal myelination) (P ≤ 0.01; Fig. 6B) compared to the contralateral naïve left nerve (LN-C). In MSC treated animals there was a significant (P < 0.001) decrease in axonal area in the right repaired tibial nerve (RN-MSC) compared to the contralateral left naïve nerve (LN-MSC) (Fig. 6A). However, the *g* ratio (axonal myelination) in the right repaired tibial nerve with MSC treatment (RN-MSC) (Fig. 6B) was not significantly different (P > 0.05) from contralateral left naïve nerve (LN-MSC).

## 4 Discussion

Our findings include early onset of sensory function, improved motor function and reduced flexion contractures with MSC administration. In INR the gastrocnemius muscle atrophy and foot flexion contractures were reduced, and functional recovery was markedly improved compared to SNR.

MSC therapy improved sensory and motor function recovery in both SNR and INR models. Goel and co-workers [7] transplanted bone marrow-derived mononuclear cells in the sciatic nerve transection model and found enhanced nerve regeneration. They attributed this effect to stem cell trophic factors causing axonal growth and stem cell differentiation into Schwann-like cells, leading to myelin reformation. Other reports have demonstrated the ability of bone marrow MSCs to differentiate into Schwann cell-like cells both in vivo and in vitro and inducing myelination of regenerated nerve fibers after sciatic nerve injury [14,51,52]. None of the above studies described studied distal branch (tibial, peroneal or sural) nerve transections. To our knowledge, ours is the first attempt where tibial, peroneal and sural nerve transections have been performed simultaneously, and repair outcomes are studied in comparison with sciatic nerve transection/repair following MSC administration.

The sensory function recovery in the nerve boundaries (tibial, peroneal, sural) observed as early as 2–4 weeks and further significant progress until about 18 weeks post nerve repair in all groups agrees with previous reports on rat sciatic nerve crush injury [48,[53], [54], [55]]. In our study, MSCs did not affect sensory function significantly. However, Song et al. observed a significant (P < 0.05) improvement in sensory function with MSC administration in hind-limb allograft model (sciatic nerve transection) in rats. To our knowledge there are no other peripheral nerve damage studies reported similar to our model where they have tested individual nerve boundaries for sensory function.

The motor function was markedly improved in INR model compared to SNR model. This was probably due to the injury in the INR model which was more distal and closer to the target of innervation. Our findings are in agreement with previous reports [[56], [57], [58]] and it has been documented that outcomes are directly related to the level of the peripheral nerve injury; distal injuries have superior functional recovery to proximal injuries. The triple nerve repair technique used in our INR model, though it requires more time and is technically more challenging, may provide superior outcomes in the context of limb transplantation. Yeh and co-workers [43] used distal nerve anastomosis techniques (tibial, peroneal, sural) which enhanced motor function in limb transplants. In our study where the nerve was completely transected and repaired (similar to a limb transplant situation), motor function improved markedly with MSC administration in both INR and SNR models. This is in agreement with the findings of Wei and co-workers [59] who used slightly different model where sciatic nerve was partially transected along a 10 mm segment and the injured site was wrapped around with a scaffold containing adipose derived stem cells; this resulted in significant improvement in motor function by 24 weeks. In sciatic nerve crush injury model, administration of human amniotic fluid stem cells [8] and adipose derived mesenchymal stem cells [17] has been shown to improve motor function (SFI) significantly. However, Song and co-workers [60] in their rat hind limb allograft model observed no difference in the motor function (SFI) recovery between bone marrow-derived mesenchymal stem cell treated and untreated groups. Nonetheless, they observed increased myelinated axons and Schwann cells. In our study motor function recovery was significantly improved in the MSC administered INR group and there was a steady ongoing clinical improvement at 18 weeks which was probably due to the proximity of nerve transection/repair to the target muscles and mesenchymal stem cell neurotrophic, anti-inflammatory and anti-apoptotic effects which have been demonstrated in previous studies. Nerve repairs in both SNR and INR models grossly looked well aligned, yet nerve function recovery was suboptimal more so in the SNR model; this was possibly due to improper axon growth, improper apposition/alignment of the nerve fascicles [6,7,51,[61], [62], [63], [64]] or other unknown factors.

Significant limb muscular atrophy in the SNR model was probably due to poor peripheral nerve regeneration and re-innervation of the target muscles and agrees with previous reports [21,22,65]. Improved motor function in our INR model and other studies [43] could be attributed to reduced target muscle atrophy. Administration of MSCs in our study did not reduce the muscular atrophy in SNR or INR models similar to Song and co-workers [60] study in rat hind-limb transplant model. However, Chen and co-workers in their sciatic nerve conduit model observed increased gastrocnemius mass and motor function recovery with bone marrow-derived stromal cell administration [14]. These variations are probably due to the differences in the models used. In a rat facial nerve (buccal branch) transection/injury model, artificial nerve conduit containing adipose-derived stromal vascular fraction (composed of MSCs, endothelial cells, pericytes, smooth muscle cells, tissue macrophages and lymphocytes) significantly improved facial nerve regeneration and facial mimetic function significantly [66]. It is probable that other cell types might be needed in conjunction with MSCs to promote muscle regeneration and/or mitigate muscular atrophy.

Furthermore, neurotrophic factors such as BDNF (brain-derived neurotrophic factor), GDNF (glial-derived neurotrophic factor) and IGF-1 (insulin-like growth factor) have well-documented effects in the peripheral nervous system [67]. Increased neurotrophic factor expression results in an increase in axon sprouting, improved regeneration of the nerve with associated greater muscle mass of the target organ and consequent accelerated recovery of motor function [68]. Granulocyte-Colony Stimulating Factor (G-CSF) can also act on neuronal cells as a neurotrophic factor; its receptors have been shown to be expressed in neurons. In several models of peripheral nerve [8] and spinal cord [69] injury, it has been demonstrated that G-CSF promotes nerve regeneration and function significantly. G-CSF has been shown to induce neurogenesis, increase neuroplasticity and counteract apoptosis. We believe that one or more of these growth factors in combination with MSCs would enrich the microenvironment and more beneficial in the treatment of peripheral nerve damage.

The limitations of the present study were: 1) Sensory function evaluation protocol we used was subjective in nature where experimenter stimulated nerve boundaries manually by pinching with forceps and recorded response. Though the method used is standard and acceptable in the field, we would have preferred having an electrostimulator to precisely stimulate nerve boundaries and measure the response; 2) Physiotherapy is an important part of the post-operative care in peripheral nerve damage and repair; we provided manual physiotherapy to rats one-two times (5 min/session) a week and housed animals in a wire mesh floor cage for about 8 h a day. It would be preferable to use an animal treadmill to provide consistent and more prolonged/frequent exercise to enhance physiotherapy; 3) We did not perform MSC homing studies as it was beyond the scope of the present study instead we relied on literature information that circulating MSCs home inflamed sites/lesions preferentially, and our protocol included local direct administration of MSCs to the target site, as well; and 4) It appears, combination cell therapy (MSCs in conjunction with other cell types or growth factors) would be beneficial to promote muscle and nerve regeneration in peripheral nerve damage but it was beyond the scope of this study to test.

## 5 Conclusion

The strategy used to improve limb function recovery in peripheral nerve damage utilizing MSCs is attractive, feasible, and promising. Limb functional recovery was superior in distal nerve injury/repair (INR) model. In a clinical transplant setting, limb amputations done as distally as possible could allow transplantation (nerve repair) closer to the target and improve functional recovery. In our study, MSC therapy improved limb functional recovery in peripheral nerve damage. Research to identify novel approaches such as stem cell therapy is expected to make a significant impact in the clinical outcome of sciatic nerve injuries and limb transplantation.

## Ethical approval

Yes. Approved by the Madigan Army Medical Institutional Animal Care and Use Committee (IACUC). Approved Protocol No. 212111.

## Sources of funding

None.

## Author contribution

Shashikumar K. Salgar: conceptualization, study design, performed experiments, data analysis & interpretation, project administration, execution & supervision, wrote manuscript draft, revised, critical review and approval of final version.

Jason R. Bingham: JRB: performed surgery & experiments, data collection & interpretation, wrote manuscript draft, and critical review & approval of final version.

Kevin R. Kniery: performed experiments, critical review of manuscript and approval of final version.

Nikolas L. Jorstad: performed histology & histomorphometry, data collection, analysis & interpretation, critical review of manuscript and approval of final version.

Iren-Horkayne Szakaly: provided histology services and reagents, performed histology, data collection & interpretation, critical review of manuscript and approval of final version.

Zachary S. Hoffer: provided histopathology support, data analysis and interpretation, critical review of manuscript and approval of final version.

## Conflicts of interest

None.

## Trial registry number

Animal studies; Not Human studies.

## Guarantor

Shashikumar K. Salgar, PhD.

Lead Research Physiologist.

Chair, Scientific Review Committee.

Department of Clinical Investigation.

Madigan Army Medical Center.

9040 Jackson Avenue.

Tacoma, Washington 98431-1100.

Phone: 253-968-6025; Fax 253-968-1044.

Email: Shashikumar.k.salgar.civ@mail.mil

## Consent

Animal studies; Not Human studies.

## Provenance and peer review

Not commissioned, externally peer reviewed.

## Potential and real conflicts of interest

The authors declare no conflict of interest.

## Disclosure

The views expressed are those of the author(s) and do not reflect the official policy of the Department of the Army, the Department of Defense or the U.S. Government.
