# Supplementary material for: “Stem cell therapy to promote limb function recovery in peripheral nerve damage in a rat model” – Experimental research
Source: Ann Med Surg (Lond). 2019 Mar 28;41:20–8. doi: 10.1016/j.amsu.2019.03.009 (PMC6463551; doi:10.1016/j.amsu.2019.03.009)
Supplement: Multimedia component 1 [file mmc1.docx]

**Table S1** Animal weight and gastrocnemius muscle weight (Mean ± SD) in Sciatic and Individual Nerve Repair Models

| Group | Animal weight at surgery (g) | Animal weight at euthanasia (g) | Naïve contralateral limb (left) Gastrocnemius muscle weight (g) | Nerve Repaired limb (right) Gastrocnemius muscle weight (g) | Gastrocnemius right : left muscle weight ratio | Percent right gastrocnemius weight compared to contralateral (left) gastrocnemius. |
| --- | --- | --- | --- | --- | --- | --- |
| SNR-Vehicle (n=12)  SNR-MSC (n=9)  INR-Vehicle (n=12)  INR-MSC (n=10) | 320.75±21.87^a^  346.89 ±20.24^a^  328.75±17.09^a^  334.50±36.38^a^ | 458.42±43.95^a^  441.33±37.52^a^  478.92±55.90^a^  459.80±36.51^a^ | 2.50±0.25^a^  2.49±0.22^a^  2.50±0.25^a^  2.46±0.22^a^ | 1.67±0.20^a*^  1.61±0.18^a*^  2.10±0.30^b*^  2.11±0.22^b*^ | 0.67±0.08^a^  0.65±0.05^a^  0.84±0.12^b^  0.86±0.07^b^ | 67%^a^  65%^a^  84%^b^  86%^b^ |

SNR, Sciatic nerve repair; INR, Individual nerve repair; MSC, Mesenchymal Stem Cells; *, Significant (P<0.05) reduction in right gastrocnemius weight compared to contralateral left gastrocnemius in all groups. Body weight at the time of surgery or euthanasia and naïve contralateral limb (left) gastrocnemius muscle weight between the groups did not vary significantly (P>0.05; shown with common superscripts in columns). Gastrocnemius weight of nerve repaired limb in INR model was significantly (P<0.05) higher (84-86% of naive gastrocnemius weight) than in SNR model (65-67% naive gastrocnemius weight); shown with uncommon superscripts. However, right gastrocnemius weight or ratio of right to left gastrocnemius did not vary significantly (P>0.05) between MSC treated and untreated (vehicle) groups in both SNR and INR models (shown with common superscripts in columns).
