# Supplementary material for: “Stem cell therapy to promote limb function recovery in peripheral nerve damage in a rat model” – Experimental research
Source: Ann Med Surg (Lond). 2019 Mar 28;41:20–8. doi: 10.1016/j.amsu.2019.03.009 (PMC6463551; doi:10.1016/j.amsu.2019.03.009)
Supplement: Multimedia component 2 [file mmc2.docx]

**Table S2** Sensory nerve function score (Mean ± SD) post-nerve repair as determined by cutaneous pain reaction (pinch reflex) test in the nerve transected and repaired foot.

| **WEEKS**  **Post-Nerve Repair** |  | **TIBIAL** | | **SAPHENOUS** | | **PERONEAL** | | **SURAL** | | **TOTAL** | |
| --- | --- | --- | --- | --- | --- | --- | --- | --- | --- | --- | --- |
|  | Model | Vehicle | MSC | Vehicle | MSC | Vehicle | MSC | Vehicle | MSC | Vehicle | MSC |
| **2**  **4**  **6**  **8**  **12**  **16** | SNR  INR  SNR  INR  SNR  INR  SNR  INR  SNR  INR  SNR  INR | 0.25±0.35  0.61±0.74  1.44±0.79  1.22±0.77  2.44±0.65  2.72±0.58  2.80±0.38  2.97±0.09  2.94±0.13  3.00±0.00  2.91±0.29  3.00±0.00 | 0.74±0.49  0.53±0.45  1.07±0.43  1.73±0.73  2.33±0.44  2.43±0.59  2.63±0.56  2.80±0.42  2.78±0.37  3.00±0.00  2.96±0.11  3.00±0.00 | 2.86±0.39  2.97±0.09  2.78±0.16  2.67±0.39  2.86±0.26  2.81±0.58  2.83±0.26  3.00±0.00  2.89±0.29  2.67±0.45  2.91±0.29  2.81±0.33 | 2.67±0.60  2.80±0.28  2.72±0.33  2.54±0.73  2.81±0.38  2.77±0.35  3.00±0.00  2.73±0.44  3.00±0.00  3.00±0.00  2.92±0.15  2.87±0.32 | 1.28±0.71  0.92±0.47  1.39±0.63  1.13±0.47  2.05±0.81  2.19±0.88  2.47±0.69  2.36±0.50  2.39±0.74  2.14±0.74  2.64±0.56  2.72±0.34 | 1.26±0.66  1.37±0.74  1.83±0.91  1.67±0.73  1.96±0.69  1.67±0.67  1.85±0.55  2.43±0.54  2.67±0.44  2.80±0.32  2.89±0.23  2.83±0.42 | 0.00±0.00  0.00±0.00  0.03±0.09  0.46±0.43  1.22±1.11  1.81±0.76  1.83±0.94  2.03±0.64  2.39±0.58  2.50±0.46  2.63±0.50  2.90±0.15 | 0.04±0.11  0.03±0.10  0.39±0.95  0.42±0.61  0.74±0.59  1.03±0.66  1.51±0.85  2.00±0.70  2.48±0.69  2.43±0.47  2.41±0.55  2.57±0.47 | 1.09±0.29^a^  1.12±0.24^a^  1.39±0.37^a^  1.36±0.27^a^  2.14±0.48^a^  2.38±0.51^a^  2.48±0.35^a^  2.59±0.21^a^  2.65±0.35^a^  2.57±0.33^a^  2.78±0.22^a^  2.86±0.13^a^ | 1.17±0.18^a^  1.18±0.25^a^  1.53±0.45^a^  1.62±0.46^a^  1.96±0.38^a^  1.97±0.39^a^  2.25±0.42^a^  2.49±0.28^a^  2.73±0.28^a^  2.81±0.15^a^  2.79±0.16^a^  2.82±0.22^a^ |

MSC, Mesenchymal Stem Cell injected group; SNR, Sciatic Nerve Repair Model; INR, Individual Nerve Repair Model. SNR Vehicle n=12, SNR MSC n=9, INR Vehicle n=12, and INR MSC n=10. Sensory nerve function score was determined based on response to pinch in the regions supplied by individual nerve (see Figure 2) and graded from 0 to 3; 0, no response; 1, mild; 2, moderate; and 3, normal. Sensory response to individual nerve (tibial, peroneal, sural) stimuli significantly (P<0.05) improved over time (2 weeks to 16 weeks) in vehicle and MSC groups in both INR and SNR models. There was no significant (P>0.05) difference in overall (Total) sensory function between vehicle and MSC treated groups in either models at any time point (shown with common superscripts).
